# Nuclear stress bodies: a potential mechanism to shut off lethal inflammatory cytokines

**DOI:** 10.1038/s41392-025-02420-7

**Published:** 2025-10-07

**Authors:** Chang H. Kim

**Affiliations:** 1 Department of Pathology, University of Michigan School of Medicine, Ann Arbor, MI USA; 2 Mary H. Weiser Food Allergy Center, University of Michigan School of Medicine, Ann Arbor, MI USA; 3 Rogel Center for Cancer Research, University of Michigan School of Medicine, Ann Arbor, MI USA

**Keywords:** Inflammation, Infectious diseases

In a recent study published in *Cell*, an in-depth study on the formation of nuclear stress bodies (nSBs) and their impact on gene expression has been reported.^1^ The system-wide role of nSBs has been a mystery so far, and the authors presented potential roles of nSBs in negatively regulating acute cytokine responses in inflammatory conditions and in protecting patients with sepsis.

nSBs are induced by heat shock, chemical, and oxidative stresses.^2^ The nSB response is one of numerous anti-stress mechanisms to regain cellular or systemic homeostasis. More specifically, nSBs are formed when cellular processes are perturbed by thermal shock or various chemicals, such as arsenite, 8-hydroxyquinoline, zinc sulfate, proteosome inhibitors, and ibuprofen. nSBs are formed by RNA-protein interactions, particularly between pericentromeric satellite III (Sat III) long non-coding RNA and heat shock transcription factor 1 (HSF1) (Fig. 1). nSBs serve as a platform to control gene expression, RNA splicing, and protein modifications to cope with stress-induced disturbances. nSBs should not be confused with other membraneless organelles such as nuclear speckles involved mainly in mRNA splicing or cytoplasmic stress granules that sequester translationally stalled mRNA and proteins for cell survival. Despite more than three decades of research, the formation and function of nSBs were incompletely understood. Liu et al. provides insightful information about the activation of nSBs and its possible role in counteracting inflammatory responses.

**Fig. 1:**
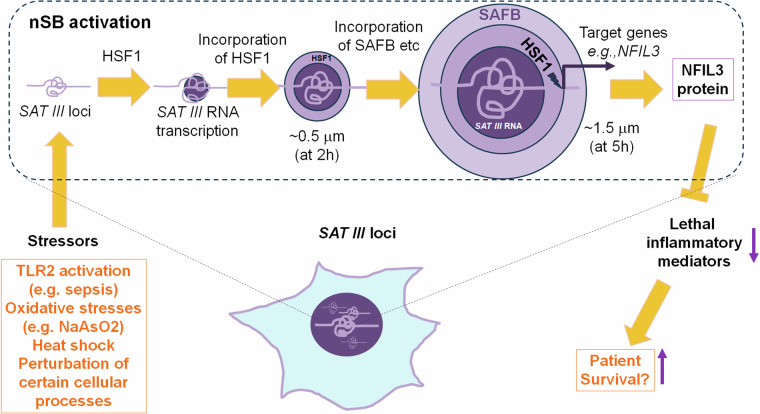
A potential role of activated nSBs in monocytic cells. At times of perturbed cellular processes induced by infections (e.g., TLR2 activation), oxidative stress, chemicals, and/or heat shock, the 13–17 *SAT III* loci across the human genome open up and rapidly expand in size within 1–2 h. Early during the activation, HSF1 is activated to bind HSEs in the *SAT III* loci to induce the production of *SAT III* RNA by RNA Polymerase II, and this process continues to expand the size of the nSB core. The produced *SAT III* RNA cores recruit HSF1 proteins and then other proteins that make up the outer layers of nSBs, all of which promote the robust expansion of nSBs close to target genes. This expansion effectively allows the transcription factors and splicing factors in nSBs, including HSF1, to act on a number of target DNA loci. One of the target genes is *NFIL3*, a gene that codes for a transcription activator and repressor protein with important functions in the immune system. The NFIL3 gene is transcribed, in part, because of the nSB expansion. Elevated levels of NFIL3 lead to active suppression of the expression of lethal inflammatory mediators (e.g., TNFα, IL-1β, and IL-8) in cells, particularly monocytic cells. Suppression of these inflammatory mediators at the systemic level has the potential to decrease the mortality of patients with sepsis and other systemic inflammatory conditions

Acute inflammatory cytokines, such as IL-1β, IL-6, TNF-α, and IL-8, are produced by various cell types including monocytes and macrophages during inflammatory or infection conditions. Sepsis conditions, caused by systemic infection by bacteria, viruses, or fungi, challenge cells with excessive activation of pattern recognition receptors and cytokine receptors. While these cytokines initiate protective immune responses by recruiting and activating immune cells, systemic production of these cytokines in large quantities can be life-threatening.^3^ Thus, mechanisms that curve the production of these cytokines would be beneficial.

Liu et al. determined the detailed process to form mature nSBs, which are composed of the *SAT III* RNA core, and the HSF1 mid-layer, and finally the Scaffold attachment factor B (SAFB) outer layer (Fig. 1). nSBs contain additional ~30 proteins, including heterochromatin Protein 1α, bromodomain-containing protein 4 (BRD4), heat shock transcription factor 2 and thyroid hormone receptor-associated protein 3, which cooperatively contribute to the structure and function of nSBs.^2^ They observed that most of the 13–19 *Sat III* loci, found primarily on the short arms of the human acrocentric chromosomes, actively produce *SAT III* RNAs under stress. Under normal conditions, HSF1 exists in an inactive monomeric form but becomes trimerized when activated by various stressors.^2^ Activated HSF1 specifically binds to heat shock DNA elements (HSEs) in *SAT III* loci for transcriptional activation. Therefore, the nSB response is considered a part of the broader HSF1 functions. Beyond the nSB response, HSF1 induces the transcription of genes that code for various proteins involved in protein folding/processing, ribosome biosynthesis, RNA metabolism, cell cycle, and inflammation. It also regulates diverse biological processes such as glucose metabolism, cell cycle progression, gametogenesis, and aging. While *SAT III* sequence repeats and nSBs are unique to primates, HSF1 is widely present in most species. HSF1 deficiency increased malignant transformation, cancer cell survival, proliferation, and metastasis. Importantly, HSF1 is implicated in suppressing the expression of the inflammasome protein NLRP3,^4^ required for production of inflammatory cytokines such as IL-1β and IL-18. Therefore, the proposed function of nSBs by Liu et al. is well within the known functions of HSF1 in regulating inflammatory responses.

An interesting observation by Liu et al. is that stressors induce rapid transcriptional activation on *SAT III* loci and enlargement of the overall nSBs structure most likely due to subsequent RNA-protein incorporation. They found that the nSB enlargement delivers key transcription inducers (e.g., HSF1 and BRD4) to neighboring genes, such as *NFIL3*. NFIL3, also called E4BP4, represses its target genes by recruiting epigenetic regulators such as histone deacetylase 2 and G9a histone methyltransferase. Increased expression of *NFIL3* but decreased expression of major inflammatory cytokines such as *IL1B1*, *IL8*, and *TNF* occurred presumably by nSB activation under stress.

Regulation of NFIL3 has many ramifications in the immune system beyond the observed suppression of inflammatory cytokines.^5^ NFIL3 is required for normal development of natural killer cells, innate lymphoid cells, macrophages, CD8α^+^ dendritic cells, and basophils. NFIL3 supports the expression of the cytokine IL-4 in T cells and binds the Igε promoter to specify immunoglobulin production in B cells. Beyond immune cells, NFIL3 regulates the circadian rhythm, hepatic metabolism, and gut microbiota.^5^ Immune dysregulation with increased numbers of functionally specialized T cells, such as Th1 cells and Th2 cells, occurs in NFIL3-deficient mice. Therefore, the positive effect of nSBs on NFIL3 expression could have far-reaching biological consequences.

Liu et al. showed that septic-like conditions created with pathogen-associated molecular patterns, particularly with the Toll-like receptor 2 (TLR2) agonist lipoteichoic acid increased *Sat III* RNA expression. This establishes TLR2 ligands (and sepsis and similar infectious conditions) as another type of nSB-inducing stressors beyond heat shock and chemical stresses. Importantly, the *Sat III* RNA expression level positively correlated with the survival rate of sepsis patients,^1^ suggesting that nSBs might have potentially protective effects in inflammatory conditions.

While the potentially important role of nSBs in counteracting the production of inflammatory mediators in physiological settings remains to be established, this opens the door for many new questions. It remains to be determined whether the observed suppressive function of nSBs can be extended to other cell types such as epithelial cells, macrophages, and lymphocytes. It remains unknown whether the suppressive effect of nSB response is functionally significant in vivo. Moreover, it is important to know if the suppression would occur selectively or conditionally, because excessive suppression of the cytokines would be also harmful for patients. The observed suppressive effect is likely to be one of numerous functions of nSBs, because nSBs contain other proteins with diverse functions. Moreover, HSF1 itself can induce many target genes beyond *NFIL3*. In this regard, the expression of *NR4A3*, *DNAJA1*, *TENT5C*, *GPBP1*, and *HSPH1* was also increased in stressed cells in a partially *Sat III* RNA-dependent manner.^1^ The mechanism for the expression of these additional genes needs to be determined. Septic shock is a complex process mediated by a myriad of microbial and host factors. There is a need to systematically study the function of nSBs in normal and various inflammatory conditions, including sepsis, inflammatory bowel diseases, and graft-versus-host-disease. Because NFIL3 has diverse functions in human physiology and immune regulation, further investigation of the pathway should yield a more complete picture of the role of nSBs.

## References

[1] Liu, X. Q. et al. De novo assembly of nuclear stress bodies rearranges and enhances NFIL3 to restrain acute inflammatory responses. *Cell* **188**, 4586–4603.e31 (2025). 40436014 10.1016/j.cell.2025.05.003

[2] Chatterjee, M., Dass, J. F. & Sengupta, S. Nuclear stress bodies: interaction of its components in oncogenic regulation. *J. Cell. Biochem.* **120**, 14700–14710 (2019). 31090102 10.1002/jcb.28731

[3] Hack, C. E., Aarden, L. A. & Thijs, L. G. Role of cytokines in sepsis. *Adv. Immunol.* **66**, 101–195 (1997). 9328641 10.1016/s0065-2776(08)60597-0

[4] Jin, Y. et al. Jagged1-mediated myeloid Notch1 signaling activates HSF1/Snail and controls NLRP3 inflammasome activation in liver inflammatory injury. *Cell. Mol. Immunol.* **17**, 1245–1256 (2020). 31673056 10.1038/s41423-019-0318-x PMC7784844

[5] Zeng, L. et al. A new border for circadian rhythm gene NFIL3 in diverse fields of cancer. *Clin. Transl. Oncol.* **25**, 1940–1948 (2023). 36788184 10.1007/s12094-023-03098-5

